# Nasoendotracheal tube obstruction by a nasal polyp in emergency oral surgery: a case report

**DOI:** 10.1186/1749-7922-2-31

**Published:** 2007-11-22

**Authors:** Tatjana Goranovic, Morena Milic, Predrag Knezevic

**Affiliations:** 1 Sveti Duh General Hospital, Department of Anaesthesia and ICU, Zagreb, Croatia; 2 Dubrava University Hospital, Department of Anaesthesiology, Reanimatology and Intensive Care Medicine, Zagreb, Croatia; 3 Dubrava University Hospital, Department of Maxillofacial and Oral Surgery, Zagreb, Croatia

## Abstract

Nasal polyps can make nasoendotracheal intubation difficult. We present a case of complete obstruction of a nasoendotracheal tube by a nasal polyp during a blind nasoendotracheal intubation in emergency oral surgery.

## Background

Nasoendotracheal tube obstruction during intubation may occur due to the turbinate destruction [1-7]. It is a rare but possible complication. Although minor mucosal trauma is quite frequent during nasal intubation, nasoendotracheal tube obstruction caused by nasal polyps has not been reported as often as expected. We present a case of a complete obstruction of the nasoendotracheal tube by a nasal polyp during blind nasoendotracheal intubation (BNTI) in emergency oral surgery.

## Case presentation

A 38-year-old man, assessed as American Society of Anesthesiologists (ASA) grade II, was admitted to our emergency department one hour after a car crash accident. On admission, the patient was conscious but amnestic for the event. His Glasgow Coma score was 15 and he complained only of pain in the mandible. Routine examinations, including cranial radiography and chest x-ray, were performed to exclude major thoracic and head injuries. A neurosurgeon confirmed cerebral concussion without neurological deficits and cervical injury. A dental x-ray revealed an open fracture of the mandible with distorted dental occlusion. Four hours after admission, the patient was scheduled for emergency fixation of the fractured mandible.

On the preoperative anesthesiologic evaluation, the patient reported to be allergic to dust and fasting for a minimum of 5 hours. He was obese (weight 100 kg; height 180 cm, body mass index 30.8 kgm^-2^), his dental caps were loose, but all routine laboratory findings were within normal range.

Anesthesia was induced with 2.5 mg of midazolam, 20 mg of etomidate, and 0.15 mg of fentanyl, followed by 100 mg of succinylcholine after 30 seconds to facilitate the intubation. When the patient was ready to be intubated, a nasal tube (size 7.5) was smoothly inserted through the right nostril into the trachea without using any force. The right position of the tube was confirmed by direct Macintosh laryngoscopy. However, once the tube was placed in the right position, manual ventilation was impossible to perform. The tube was immediately removed and the diagnosis made on-site: a nasal polyp completely obstructed the lumen of the tube and was still partly protruding from its tip. The second attempt of nasal intubation was successfully performed through the left nostril. The surgery was completed without complications. The patient was admitted to the intensive care unit (ICU) for observation and extubated. The following day, he was transferred from the ICU to the Maxillofacial Ward and discharged in good condition from the hospital 7 days later.

## Discussion

Intranasal deformities were reported in over two-thirds of oral surgery patients, although they had patent nostrils on clinical examination and no history of nasal obstruction [8]. The most common abnormality was a deviated nasal septum, whereas polyps as an obstructive abnormality were found in only 2% of the cases [8].

Nasal polyps are non-neoplastic masses of edematous nasal or sinus mucosa. Different causes of nasal polyps require both medical and surgical therapy [9]. Prevalence is generally reported between 1–4% with higher prevalence in patients with asthma and cystic fibrosis. According to Danish authors the overall estimated incidence of symptomatic nasal polyps was 0.627 patients per thousand per year [10].

Given that these finding refer to elective cases and that many intranasal abnormalities can even then be easily neglected if endoscopic examination is not performed, emergency situations call for extra caution. In emergency cases, a tendency to speed up the physical examination and history taking carries the risk of overlooking the history of intranasal deformities. Furthermore, in oral surgery cases, the nature and site of trauma often affect the present intranasal deformities, making the nasal intubation more difficult, although it is a preferable type of intubation in oral surgery.

Unlike the turbinate, nasal polyps are soft tissue growths and can easily be amputated during trauma. Therefore, it is possible that in our patient the nasoendotracheal tube could smoothly pass through the nasal cavity and be inserted in the trachea without the polyp impeding the passage because it was amputated. On the other had, it obstructed the tip of the tube.

## Conclusion

Nasoendotracheal tube obstruction during BNTI may be caused by the nasal polyp. When carrying out emergency BNTI and relying only on patient history, anesthesiologists should be aware of this rare complication and perform manual ventilation after intubation very gently in order not to push the amputated soft tissue growth further down the trachea or bronchi.

## Competing interests

The author(s) declare that they have no competing interests.

## Authors' contributions

TG was the anesthesiologist in the described case and drafted the manuscript.

MM and PK performed the literature search and jointly wrote the manuscript.

All authors read and approved the submitted version of the manuscript.
